# Aligning Patient’s Ideas of a Good Life with Medically Indicated Therapies in Geriatric Rehabilitation Using Smart Sensors

**DOI:** 10.3390/s21248479

**Published:** 2021-12-19

**Authors:** Cristian Timmermann, Frank Ursin, Christopher Predel, Florian Steger

**Affiliations:** 1 Institute of the History, Philosophy and Ethics of Medicine, Ulm University, 89073 Ulm, Germany; christopher.predel@uni-ulm.de (C.P.); florian.steger@uni-ulm.de (F.S.); 2 Institute for Ethics, History and Philosophy of Medicine, Hannover Medical School, 30167 Hannover, Germany; ursin.frank@mh-hannover.de

**Keywords:** digitalization, older adults, wearable sensors, intelligent sensors, healthcare, capabilities approach, therapy, ethics

## Abstract

New technologies such as smart sensors improve rehabilitation processes and thereby increase older adults’ capabilities to participate in social life, leading to direct physical and mental health benefits. Wearable smart sensors for home use have the additional advantage of monitoring day-to-day activities and thereby identifying rehabilitation progress and needs. However, identifying and selecting rehabilitation priorities is ethically challenging because physicians, therapists, and caregivers may impose their own personal values leading to paternalism. Therefore, we develop a discussion template consisting of a series of adaptable questions for the patient–physician encounter based on the capability approach. The goal is to improve geriatric rehabilitation and thereby increase participation in social life and well-being. To achieve this goal, we first analyzed what is considered important for participation on basis of the capability approach, human rights, and ethics of care. Second, we conducted an ethical analysis of each of the four identified dimensions of participation: political, economic, socio-cultural, and care. To improve compliance with rehabilitation measures, health professionals must align rehabilitation measures in an open dialogue with the patient’s aspiration for participation in each dimension. A discussion template based on the capability approach allows for a proactive approach in patient information and stimulates a critical assessment of treatment alternatives while reducing the risk of imposing personal values.

## 1. Introduction

Mobility is generally seen as a prerequisite to participate in a wider range of social, cultural, economic, and religious activities. To make sure older adults have the opportunity to participate in the diverse activities that constitute a good life, it is imperative to improve their mobility and reduce hurdles to access public sites. New technologies such as smart sensors promise to improve rehabilitation processes. Smart sensors are devices that measure and analyze specific patterns and have the capacity to communicate this data to a sensing network [1]. The aim of the current work is to provide ethical guidance to health professionals on how to discuss with their patients the rehabilitation options involving smart sensors.

Traditional hospital-based rehabilitation comes with significant challenges, such as allowing older adults to maintain their daily routines, the hassle of commuting to and from the site, and providing person-centered therapy. New technologies, such as smart sensors (see Box 1 for an overview) can be used at home and thereby alleviate these challenges. Smart sensors permit user-driven and participatory implementation of geriatric rehabilitation technologies [2]. Technology developers have managed to reduce the size of many of such sensors so that they can be worn comfortably to track daily activities while collecting important data. This also allows a certain degree of discretion to avoid possible stigmas of wearing medical devices [3]. The added value of smart sensors within the group of wearables is that they not only collect data but also interpret it by identifying patterns in the data, thereby providing important information to health professionals on rehabilitation progress and risk factors. Machine learning algorithms as a new technological opportunity allow an extensive collection and in-depth analysis of data [4]. However, the effective use of smart sensors and the success of long-lasting rehabilitation therapies depend largely on patient compliance, as patients themselves can decide on whether and when to wear the sensors [5].

To increase compliance, physicians and therapists need to align the choice of rehabilitation measures to the patient’s goals in regaining participation. Working with older adults brings two major challenges, which are particularly prevalent in this age group (70+ years). Due to their advanced age, it is likely that many older adults have accepted their mobility restrictions and adapted their treatment expectations accordingly. Furthermore, some older adults may have a low sense of self-worth [6] and find their situation hopeless [7], which may make the patient information process and adherence to long-term rehabilitation therapies particularly challenging. Older adults who may not be able to care for themselves and for others, nor participate in the activities they value, may have difficulties in accepting their position of dependence, particularly in cases where they personally feel that they cannot reciprocate in any meaningful way. It is therefore imperative to expand the opportunities of participation in social activities and offer new forms of interaction with others to improve quality of life and mental health. As self-evaluation of health conditions might be deeply anchored and difficult to change, health professionals will have to discuss treatment options proactively to nudge older patients to evaluate such options in view of the multiple advantages of a successful rehabilitation. A risk of such a proactive approach is the imposition of personal values and paternalism [8].

To reduce the negative effects of such an approach, we propose the use of a discussion template on the basis of a simplified version of the ten Central Human Capabilities by Martha Nussbaum [9]. This approach concentrates on what people commonly value to do and be, based on philosophical and historical analysis as well as empirical observations [10]. A discussion template can assist health professionals in guiding a conversation to identify the issues patients may value doing or being. The proposed template consists of a series of semi-structured questions regarding different dimensions of participation. By discussing widely shared ideas of a good life, physicians, therapists, and caregivers can recognize their own biases and patients can re-evaluate their own condition and expectations. The aim of this research is to develop such a discussion template to empower patients to decide on the level of monitoring they should be subject to in line with their own rehabilitation goals.

Box 1 **Use cases of smart sensor technologies for geriatric rehabilitation.**

Smart sensors combine the measurement and analysis of data. Depending on the type of sensor, they can collect a wide variety of data [11]. In this article, we focus on smart sensors that collect data and use machine learning algorithms to detect and identify specific movement patterns. Patients can wear smart sensors at home. Thus, by using smart sensors the activities of daily living and the progress of rehabilitation can be objectively assessed and improved [12,13]. Smart sensors in conjunction with apps are developed for certain somatic affections:

- Parkinson’s disease: The Gait Tutor (mHealth Technologies) is a commercially available medical device that includes a smartphone application and gait sensors. The device assesses gait quality in real time and gives voice instructions for a safe, effective, and steady gait. This enables gait rehabilitation for, e.g., Parkinson’s patients without the patients having to attend a clinical facility [14]. The accuracy and precision is as good as in professional gait labs [15].
- Cardiac rehabilitation: A system consisting in a wearable device and a smartphone application measures and reports instantaneous heart rates during intensive physical exercise. The smartphone application is connected to a web interface and a database at a medical station to monitor the prescribed exercises. The system enables patients to conduct their rehabilitation at home instead of a hospital [16]. It eliminates the need for self-reporting, which can be prone to bias.
- Pulmonary rehabilitation: A remote system for a multimodal sensors-based application provides a cost-effective rehabilitation at home. Patients who have chronic breathing difficulties can thereby track their progress and performance and receive exercise assignments and guidance [17].

## 2. Materials and Methods

**Figure 1:**
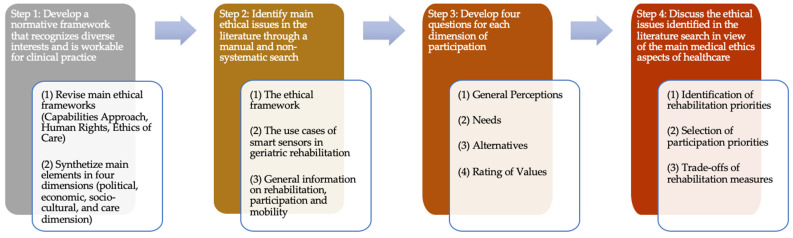
The 4 Steps to align patient’s ideas of a good life with medically indicated therapies.

This article focusses on older adults seeking to partly or fully recover their mobility and thereby increase their participation. We proceeded in four steps to develop a discussion template for aligning patients’ participation goals with medically indicated rehabilitation measures using smart sensors (Figure 1).

**Figure 2:**
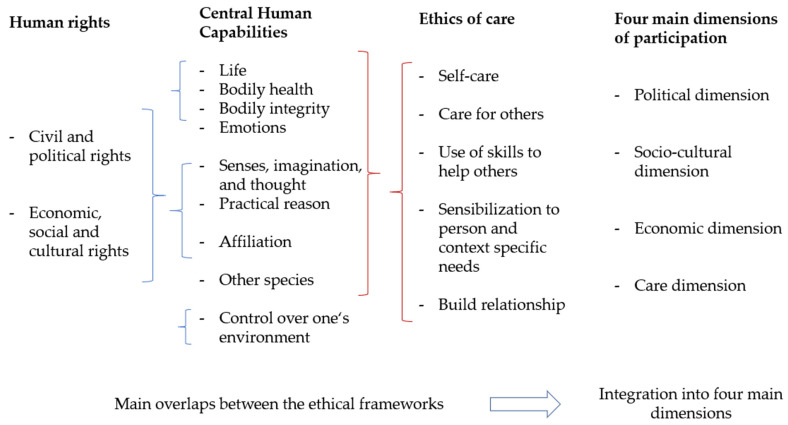
Development of the ethical framework.

**Figure 3:**
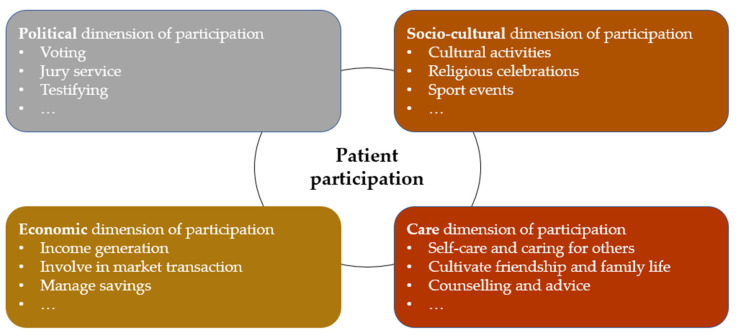
Four dimensions of participation often limited by mobility restrictions.

First, to identify what people value to be or do, we chose to base our ethical analysis on the capabilities approach [10]. The capabilities approach is a widely used ethical approach to identify central human interests and has been applied to the design of technologies more broadly, i.e., capability-sensitive design [18,19], and as an argument for patient-centered care more specifically [20]. To make this approach more accessible to healthcare professionals, we decided to align the main findings of the capabilities literature with the more commonly known human rights language [21]. This has the advantage of not only appealing to moral rights but also to legal rights, particularly within jurisdictions that have incorporated human rights in their national laws. To address feminist critiques to the human rights discourse, we complement our approach with the major insights of ethics of care to reduce important omissions. We therefore searched three ethical frameworks for the dimensions that are generally considered important for participation: the capability approach, human rights, and ethics of care. For this purpose three major sources were examined: (i) Martha Nussbaum’s list of central human capabilities [9], (ii) the International Covenant on Civil and Political Rights (1966) and the International Covenant on Economic, Social and Cultural Rights (1966), and (iii) Eva Kittay’s ethics of care [22]. Taking smart sensors in geriatric rehabilitation as a case in point, we thereby identified four major dimensions of participation that older adults may value based on the interests of the general population: political, economic, socio-cultural, and care dimensions (Figure 2). By concentrating on four dimensions, we strike a balance between proposing a framework that recognizes diverse interests and a tool that is still workable for clinical practice. We screened the abovementioned literature (i–iii) for main overlaps and justifications on why the respective dimension is deemed important, especially for older adults. Due to the fact that personal preferences regarding specific activities in the four dimensions of participation differ due to cultural peculiarities and social infrastructure, as well as individual factors, such as motivation and prejudices [23], we provide only exemplary activities on a high level of aggregation (Figure 3).

Second, we conducted three manual and non-systematic Google Scholar searches on the following themes: (a) For the ethical frameworks we searched with the strings (“capability approach” OR “capabilities approach” AND “older adults”), (“human rights” AND “older adults”), and (“ethics of care” AND “older adults”). We considered only the first 50 hits (sorted by relevance) and included only those publications that specified issues related to participation. We stopped including publications when each of the four dimensions was saturated for our purpose of specifying the theoretical framework. This allowed for the elaboration of the ethical implications of expanding patient participation when using smart sensors in geriatric rehabilitation by addressing the following question in the discussion of this article: What are the ethically relevant issues when patients use or do not use smart sensors for rehabilitation measures? (b) For specific use cases of smart sensors in geriatric rehabilitation (see Box 1), we searched manually with combinations of the strings “smart sensors”, “wearables”, “rehabilitation”, and “older adults” also following citation chains. (c) The third thematic search on “rehabilitation”, “participation”, “mobility” aimed at informing the discussion of this work. After screening titles and abstracts of the first 50 hits in the searches (b) and (c), we primarily included publications that addressed ethical aspects.

**Figure 4:**
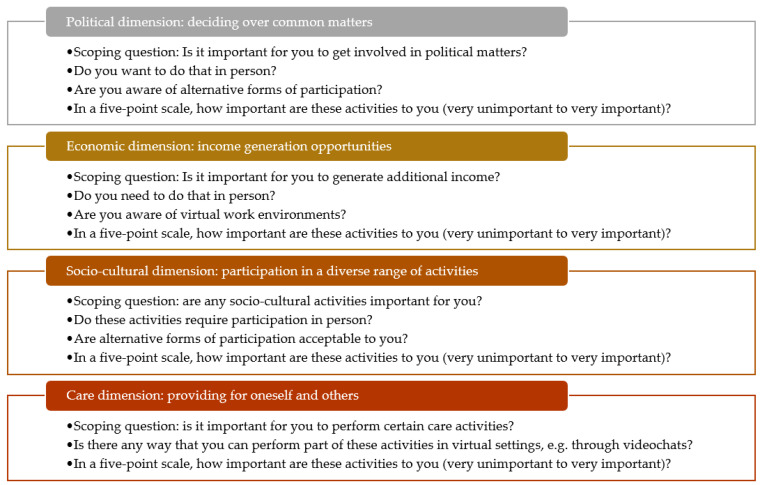
A proposal for a discussion template informed by the capability approach.

Third, we developed four questions to encourage conscious reflection of patients for each of the four dimensions of participation, which have been identified in the first step (Figure 4). The questions are not intended to scientifically measure patients’ attitudes towards politics, economy, etc., but to trigger a narrative reflection on their personal priorities with an ad-hoc assessment based on their personal values. The questions aim at perceptions, needs, alternatives, and a rating of values. As informed by the capabilities approach, our first question is a scoping question to identify whether each dimension is valued by the individual patient. The second question asks whether the patient needs or wants to participate in a particular activity in person, because this is crucial for the estimation of how much physical mobility needs to be improved. The third question asks about the awareness of alternatives to personal participation, because digital applications can substitute physical presence today. Finally, the fourth question asks for rating the importance of the respective dimensions of participation on a five-point Likert scale, because this rating tool is well suited to teasing out evaluative attitudes [24].

Fourth, we discuss the ethical implications identified in the second step against three medical ethics aspects of healthcare: identification of rehabilitation priorities, selection of participation priorities, and trade-offs of rehabilitation measures.

## 3. Results

We identified four dimensions where participation is regularly sought: political, socio-cultural, economic, and care (Figure 3). In the political dimension, people may want to seek influence on political decisions through voting, testifying, and organizing assemblies. The economic dimension may include several activities to generate income for oneself and others. The broadest dimension concerns participation in socio-cultural activities, with interests as varied as taking part in religious celebrations, watching sport events with fellow team supporters, visiting museums, or becoming involved in citizen science projects. The care dimension includes various activities to care for oneself and others through support, counselling, or company. While the significance of each person attributes to these dimensions varies widely, it is important to recognize that quality of life depends heavily on whether people are able to participate in activities they value. In addition, quality of life also depends on how engaging in these activities allows people to identify with certain ideals, such as being a caring grandfather or an engaged citizen [9].

### 3.1. Political Dimension

Securing participation in political decision making to gain control over one’s environment is seen as a central human capability [9]. It is necessary for the right to participate in public affairs [25]. For political participation to be meaningful, it is required that participation is effective and that it should have representative influence within the boundaries of democratic decision making in public affairs. Moreover, people should have adequate access to political decision-making mechanisms and public service. Any restrictions need to be well justified and alternative participation channels need to be provided to make political processes as inclusive as possible.

It is well recognized that mobility restrictions need to be addressed to secure political participation. In the case of voting, many countries offer alternatives that allow for participation with mobility restrictions. The rationale behind such accommodations is that, regardless of whether groups are large enough to influence an electoral outcome, participation is of intrinsic value for democracy and voters. In countries with a jury system, equal respect requires to make reasonable accommodations to facilitate participation for people with mobility restrictions [26]. Another important aspect is to be able to serve as a witness and to testify certain events and experiences. Possible biases may make it difficult or impossible for guardians to provide an adequate narration of an event on other people’s behalf, particularly in cases of abuse and other traumatic experiences [26].

Although there are vast discrepancies between countries, there is substantial progress towards allowing people with mobility restrictions to participate in political matters through different formats, e.g., mail, telephone, and internet [27].

### 3.2. Economic Dimension

Independent from the level of pensions and savings, another widely held interest is to generate income to improve one’s own position and the situation of loved ones. Justice requires establishing an institutional order in which people are free to work towards improving their own condition [28]. In a world where purchasing power has such a huge effect on what one is able to do, participating in economic activities has a direct relation to the capability to gain control over one’s environment [9]. Remunerated work that is meaningful also allows one to make use of a number of capabilities for a societal purpose [29,30]. Depending on the work environment, people can exercise their capabilities of “senses, imagination, and thought”, “emotions”, “practical reason”, and “affiliation” [9]. Human rights mention explicitly a “right of everyone to the opportunity to gain his living by work which he freely chooses or accepts” [31], further specifying that work environments should be safe and provide fair wages, equal opportunity, and a reasonable limitation of working hours.

Having reached retirement age does not extinguish such demands. Retirement age is nonetheless an important indicator to judge how voluntary work arrangements are. Employers and governments still need to make sure they do their best efforts in making work environments accessible to older adults and allow alternative working arrangements that benefit people with different needs and interests.

As the COVID-19 pandemic has shown, we can observe major class issues between work arrangements that allow distance participation and work that does not. While some activities are impossible to be carried out at distance, insufficient digital literacy, power disputes, and weak labor protection laws can also impede work being done remotely. Facilitating meaningful distance work will have to go hand in hand with widening training opportunities and improving labor protection [32].

### 3.3. Socio-Cultural Dimension

There are substantial differences in how much people value participating in cultural, scientific, religious, and sport activities and events. The ceremonial nature of many religious practices foresees participation in person. Similarly, as in the economic dimension, participation in socio-cultural activities allows one to exercise various capabilities. Participation in cultural, educational, and scientific events and projects is also a protected human right [31]. From an ethical perspective, it is important to note that people may have an interest in participating for instrumental reasons (i.e., to achieve a specific goal) and for intrinsic reasons (e.g., for the joy of it, collegiality, and curiosity).

When it comes to older adults, there are ample opportunities to participate in socio-cultural activities. Many institutions offer discounts to make sure older adults can participate despite low pensions. Physical access remains a problem, particularly in cities that do not provide clear walkways and accessible routes. Here it needs to be noted that most participation possibilities are restricted to passive participation, for example, being a spectator. Active participation remains rudimentary when the intellectual and physical contribution of senior citizens is not sufficiently valued.

In recent decades, we can witness a strong movement to eliminate any barriers that may hinder participating in cultural and scientific life, supported by international organizations such as UNESCO [33]. Older adults who are digitally literate and have a computer with internet connection have access to a wide range of media and documents that are openly accessible.

### 3.4. Care Dimension

Participation in care activities for themselves (self-care) and others has a special value. Providing care for others is an unavoidable element of life; if people refuse to provide such care, others need to do their share [22]. Engaging in care activities also allows one to practice several capabilities in a meaningful way, such as “senses, imagination, and thought”, “emotions”, “practical reason”, “affiliation”, and in some cases to interact with “other species” [9]. Furthermore, being able to care for oneself allows one to protect one’s life, bodily health, and bodily integrity [9]. As quality care involves the use of a wide range of capabilities, people may place a particular value in care meeting a standard and it being done under special relationships, something that cannot be carried out by others [34].

The need and desire to care for others varies depending on personal context. Older adults may want to show special dedication to their partners, grandchildren, and children but may also direct the desire to care for pets, wild animals, or plants. Participation in care activities, which often require direct interaction with people to assess neediness and emotions, and in many instances physical contact [35], is severely limited by mobility restrictions.

### 3.5. Assessing Patient’s Priorities

To personalize rehabilitation measures, the four dimensions of participation can shine light on different aspects of life where participation is sought. A discussion template based on a series of adaptable questions can pinpoint how much patients value certain areas of life [36] and suggest alternative participation methods for those areas where in person presence is not fundamental. We propose a discussion template (Figure 4), which health professionals can adapt according to their clinical experience and local circumstances.

**Figure 5:**
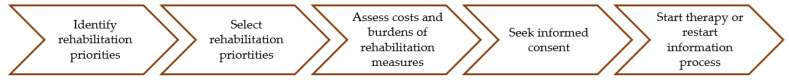
Process from identification, selection, cost and burden assessment, to seeking informed consent and starting the rehabilitation process.

These sets of questions can be used to identify rehabilitation priorities. After a careful identification and evaluation of the different priorities, the physician can guide the patient in selecting the rehabilitation priorities that are most important for the patient and have a realistic chance of success from a therapeutic perspective. After the choices have been made, the patient needs to be informed about the cost and burdens of the rehabilitation measures to obtain informed consent. In the case that the patient is informed and agrees to the rehabilitation measures and goals the therapy can begin, otherwise the information process needs to restart with a more extensive discussion of the options and goals (Figure 5).

## 4. Discussion

Increasing mobility among older adults is essential to improving their quality of life and allowing them to interact in person with other senior citizens living outside their residence and with people from other age groups. There are also direct benefits for mental and physical health. Movement permits individuals to regain independence, reduces isolation, promotes a positive mood, reduces risks, and maintains cognitive and functional ability [37]. Mobility restrictions make it even more difficult to confront loneliness by seeking outside activities and adapting socialization needs to personal preferences. As aging is the highest risk factor to health [38], rehabilitation needs increase as people age. Home-based rehabilitation systems can accelerate recovery [39], but incorrect exercises can be counterproductive or lead to injuries [40]. This makes it crucial to offer ambulatory rehabilitation therapies and improve patient information and compliance. Smart sensors provide the opportunity to measure the activity of the patient at home and to track the rehabilitation process [12]. These types of tools can improve the decision-making process between medical professionals and patients [41]. We proceed with a discussion of the main ethical challenges in using a capability approach inspired discussion template in clinical practice: (i) identification of rehabilitation priorities, (ii) assessment of alternative forms of participation, and (iii) ethical implications for privacy and accessibility.

### 4.1. Identification of Rehabilitation Priorities

As rehabilitation measures take more time and are less successful with age, it is important that rehabilitation goals are well chosen and concentrate on smaller, realistically achievable steps. Therefore, to allow patients to participate in the different political, economic, socio-cultural, and care dimensions, rehabilitation measures need to be specifically tailored to the priorities of patients. To identify the patient’s goals, a conversational approach is recommended. Instead of focusing on seeking informed consent for the rehabilitation measure that is from a medical perspective seen as the best option, a patient-physician dialogue informed by the capability approach aims to match the rehabilitation measures with the patient’s feasible mobility recovery goals, increasing both autonomy and compliance. An informed patient that can choose the rehabilitation trajectories that best match their participation aims can get the most out of the application of smart sensors.

Nonetheless, identifying the most suitable treatment options is a difficult task, because patients’ priorities vary depending on their individual circumstances and preferences [42]. In addition, patients may only voice the preferences they consider as reasonable. This may lead to the problem of adaptive preferences. As a coping mechanism, under distress people adapt their preferences to goals they consider achievable [43]. For privileged people—in terms of health and wealth—these goals may be placed beyond what can be realistically achieved. For people who had a more difficult life, these goals may be well below of what can be realized.

Although our template can help identify much broader goals and invite patients to assess areas of their lives to which they might have given little thought during a medical appointment, two concerns remain. One problem is that physicians may still insist on treatment options that are influenced by prior successes and experiences. As prior experience with a new technology is likely to be based on extreme early cases, it can be affected by the problem of path dependency, i.e., to base future development on what was technologically feasible and a priority during early stages. If early work involved mostly richer men, we can expect gender and class biases [44,45]. Physicians need to be aware of such biases in order to really be open to discussing different life plans and conceptions of a good life, particularly in regard to desired forms of interaction with others. As a second problem, the proactive handling of patient information presented here can have a directive character. When people have lowered their expectations of what they think they can reasonably achieve, it is difficult to avoid being dominant in nudging patients to critically assess the options and recognize their advantages.

Smart sensors can be used to identify activities that the patient is currently not capable of and that could be improved by rehabilitation measures. Sensors used at home can objectively reveal the activities that patients recurrently aim to achieve in daily life.

### 4.2. Assessment of Alternative Forms of Participation

As full rehabilitation might not be achievable due to advanced age, a certain prioritization of rehabilitation goals is necessary. If the ultimate goal is to expand participation in the multiple dimensions that constitute a good life, older adults need to be aware of not only options to participate in person, but also the possibilities to take part virtually. A specially trained social worker may provide important assistance in this process, as information on virtual participation is outside the standard medical curriculum. Due to the multiple mental health benefits of participating in social activities, increasing older adults’ participation through digital tools can be seen as a public health measure.

A careful consideration of the benefits and disadvantages of the different forms of participation may help patients to select the rehabilitation measures in view of expanding their capabilities set in all the dimensions of life they value. The physician can then refer the patient to a dedicated social worker who could guide the older adult to identify alternatives where virtual participation is seen as an adequate replacement. After the COVID-19 experience, it has become clear that such digital alternatives are not always an acceptable replacement, but the same experience has shown us that for many issues we did not expect earlier, online participation is a welcomed option or even a new preference. These options also reduce the risk that the patient sacrifices too much towards regaining mobility.

### 4.3. Ethical Implications for Privacy and Accessibility

The use of smart sensors gives much greater freedom to adapt rehabilitation measures to the patient’s preferences. Smart sensors can be used outside of the clinical setting, which facilitates patient compliance and gives a broader dataset for physicians to analyze rehabilitation progress. Rehabilitative measures and monitoring that previously were only performed in the hospital can be extended to provide measurements outside therapeutic settings [46]. This gives the opportunity to identify the need for preventive steps and adverse events in a daily life context [47].

A template based on the capability approach can show patients why it is good for them to follow a line of therapy, but it does not highlight the costs of movement monitoring, particularly in regard to privacy loss. An over-enthusiasm to regain mobility may reduce judgement capacities on issues of privacy. At the same time there are limits on how much physicians should insist on patients considering issues of privacy without being paternalistic.

Which safeguards should be implemented to make sure patients do not sacrifice important interests needlessly to regain mobility? Value-sensitive design has taught us that people may have various conflicting values that need to be considered in the design process [48]. The use of data-collecting sensors brings in challenges at an epistemic level, making it difficult to explain to patients the risks, costs, and benefits of such technology [49]. A general understanding is a prerequisite for informed consent. There is insufficient certainty on the amount of information that can be identified through smart sensors. Certain movement patterns may be identified in the aftermath as part of a routine [50]. 

Let us consider an example. A specific movement involving both hands at a recurrent time of the day and week may invite speculation: is the patient opening a bottle of wine? How are such incidental findings to be handled? There might be conflicting responsibilities. Physicians may ask themselves whether to respect the patient’s privacy or approach them on a potentially irresponsible conduct. For instance, what should be done when discovering that the patient is drinking while on medications that should not be taken with alcoholic beverages? The physician will have to balance considerations for respecting the patient’s requests for privacy with preventing harm. Such an assessment cannot be done with the patient without discussing issues that the patient wanted to keep private and therefore will likely not be based on the patient’s values. There are also conflicting responsibilities of physicians to society: to report potential threats that may lead to the loss of a driver’s license. Empirical studies have revealed that older adults are very open to be monitored through devices in order to avoid the massive privacy loss involved in living in a care facility [49].

Securing fair access to digital technologies is also a complex undertaking. Many older adults would need some type of subvention to secure physical access to smart sensors. In addition, technology developers will have to work with a sufficiently diverse set of patients to ensure that older adults are not negatively affected by biases and errors in the algorithms used in these sensors [45]. Lastly, smart sensors need to be designed in such a way that they are not too difficult to use for people with low digital skills and special training needs to be offered to those older adults who need to bridge this knowledge gap [51,52].

### 4.4. Limitations

This paper provides a discussion template for patients and physicians that plan to use smart sensors in rehabilitation. Prior work concentrates on the ethical analysis of sensors used in dementia care or sensors used by a younger patient group [53,54]. A limitation of this work is the low number of empirical studies that are available to assess the feasibility of using such discussion templates for complex technologies among older adults. As smart sensors are data-intensive sensory tools and the education of patients takes much time, most clinicians may have little time to discuss the ethical aspects with the patient as provided in this article in such detail. Additionally, clinicians that have received only rudimentary ethical training may have difficulties in drawing proper conclusions from the patient’s answers to the suggested questions. Further empirical studies need to explore additional preferences and concerns of patients using smart sensors. By applying qualitative methods, further research could examine how health professionals assess the overall benefits of using such devices.

## 5. Conclusions

An extensive dialogue between patients and health professionals can identify future rehabilitation strategies and develops patient support and compliance with sensor monitoring by linking rehabilitation measures with achievable future activities. Depending on their ideas of a good life, older adults may decide on different rehabilitation priorities, e.g., to recover walking abilities to enjoy the woods or arm movement to play chess with their grandchildren, to which monitoring needs to be adapted. Due to the limited success of rehabilitation measures at old age and a wide prevalence of apathy, it is important that older adults are well informed about their options and alternatives and are encouraged to critically evaluate them. Tools, such as the proposed discussion template, can support individualized evaluation of the available options by allowing health professionals to take a proactive approach in patient information, while at the same time reducing the risk of imposing personal values. For such tools to be effective, health professionals need to gain a wider understanding of the multiple components that may contribute to a good life. Proactive patient information requires a deep commitment from the part of health professionals to expand their ethical education and learn about different worldviews and life goals. A special challenge is the communication of privacy concerns that come with the use of smart sensors.

To make sure older adults can indeed benefit from smart sensors, the three major hurdles for accessibility need to be addressed. Older adults need to be supported to overcome financial constraints to access these technologies, technology developers need to make sure they work with a diverse enough set of training data to minimize potential biases, and patient education needs to help bridge eventual barriers insufficient digital literacy may pose.

Lastly, the participation of older adults in the multiple spheres of public life needs to be increased as a public health concern. Such an effort goes beyond the medical task of increasing mobility by improving rehabilitation measures. Public health officials need to work towards making the different areas of life more inclusive by making them more accessible to people with mobility restrictions and by offering additional opportunities to participate in events through digital technologies.

## Data Availability

No additional data is available.
